# Pre‐clinical development of AVB‐101, an AAV gene therapy treatment for frontotemporal dementia with progranulin mutations

**DOI:** 10.1002/alz.086546

**Published:** 2025-01-03

**Authors:** Youn Bok Lee, Carlos J Miranda, Joseph Allison, Aleksandra Kaliszewska, Yalem Bekele, Paniz Hosseini, Romain Joubert, Zoe Walker, Joana Furtado, McQuinn Gumus, Alinda Fernandes, Olivier Brock, Page Bouchard, Alex Bloom, Do Young Lee, Christopher E Shaw

**Affiliations:** ^1^ United Kingdom Dementia Research Institute, London United Kingdom; ^2^ AviadoBio, London, London United Kingdom; ^3^ United Kingdom Dementia Research Institute, Department of Basic and Clinical Neuroscience, Institute of Psychiatry, Psychology and Neuroscience, King’s College London, London, London United Kingdom; ^4^ Rising Tide Advisors, LLC, Andover, MA USA

## Abstract

**Background:**

Frontotemporal dementia (FTD) presents with a change in personality, behaviour and language and is the second most common cause of young‐onset dementia after Alzheimer’s disease. Loss of function mutations in *GRN*, encoding progranulin (PGRN), causes FTD in the heterozygous state, accounting for 5‐10% of all FTD cases. PGRN is essential for normal lysosomal function and neuronal survival. The PGRN reduction observed in FTD‐GRN patients leads to lysosomal dysfunction, associated with the mis‐accumulation of neuronal TDP‐43 and exaggerated microglial reactivity that accelerates neurodegeneration. FTD‐GRN represents a serious condition with significant unmet needs. AVB‐101 is being developed as a one‐time treatment for FTD‐GRN administered bilaterally into the thalamus (ITM) by convection‐enhanced delivery using a stereotaxic neurosurgical procedure.

**Methods:**

AVB‐101, a recombinant AAV9 vector, was designed to minimise vector dose and to restrict PGRN expression to neurons by engineering a cassette that includes (i) a codon‐optimised *GRN* sequence, (ii) PGRN expression enhancers and (iii) a neuron‐specific promoter. *In vitro* experimental testing of the construct was initially conducted in neuronal cultures. Efficacy studies were conducted in *Grn* knockout mice. Biodistribution studies were conducted first in sheep to optimize the route of administration and extended to non‐human primates (NHPs) to assess the safety and tolerability of AVB‐101.

**Results:**

AVB‐101 induces robust levels of PGRN expression in neurons when tested *in vitro*. *In vivo*, AVB‐101: (a) can suppress neuronal lipofuscinosis even at the lowest dose tested in *Grn* null mice; (b) in sheep resulted in dose‐dependent and widespread cortical and basal ganglia PGRN expression even at doses lower than commonly used with direct cerebrospinal fluid delivery modalities; (c) results in no expression of PGRN outside the CNS regardless of the animal model used; (d) in NHPs was well tolerated, with no mortality or clinically evident adverse effects; (e) in sheep and NHP drives PGRN expression to human‐equivalent physiological levels in the temporal and frontal lobes, which are the cortical regions most severely affected in FTD‐GRN.

**Conclusions:**

The preclinical data suggest that intrathalamic infusion of AVB‐101 constitutes a novel and promising approach to supplement PGRN in the CNS, supporting the clinical development of AVB‐101 for FTD‐GRN.

